# BNT162b2 COVID-19 vaccination uptake, safety, effectiveness and waning in children and young people aged 12–17 years in Scotland

**DOI:** 10.1016/j.lanepe.2022.100513

**Published:** 2022-09-28

**Authors:** Igor Rudan, Tristan Millington, Karen Antal, Zoe Grange, Lynda Fenton, Christopher Sullivan, Audrey Buelo, Rachael Wood, Lana Woolford, Olivia V. Swann, Josephine L.K. Murray, Lucy A. Cullen, Emily Moore, Fasih Haider, Fatima Almaghrabi, Jim McMenamin, Utkarsh Agrawal, Syed Ahmar Shah, Steven Kerr, Colin R. Simpson, Srinivasa Vittal Katikireddi, Sir Lewis D. Ritchie, Chris Robertson, Sir Aziz Sheikh

**Affiliations:** a Usher Institute, The University of Edinburgh, Edinburgh, UK; b Public Health Scotland, Glasgow, UK; c Department of Child Life and Health, University of Edinburgh, Edinburgh, UK; d Royal Hospital for Sick Children, Paediatric Infectious Diseases, Edinburgh, UK; e School of Medicine, University of St Andrews, St Andrews, UK; f School of Health, Wellington Faculty of Health, Victoria University of Wellington, Wellington, New Zealand; g MRC/CSO Social & Public Health Sciences Unit, University of Glasgow, Glasgow, UK; h Academic Primary Care, University of Aberdeen, Aberdeen, UK; i Department of Mathematics and Statistics, University of Strathclyde, Glasgow, UK

**Keywords:** COVID-19, BNT162b2 COVID-19 vaccination, Vaccine uptake, Vaccine safety, Vaccine effectiveness, Vaccine waning, Children and young people, Age group 12-15 years, Age group 16-17 years, Scotland, United Kingdom, National prospective cohort study

## Abstract

**Background:**

The two-dose BNT162b2 (Pfizer-BioNTech) vaccine has demonstrated high efficacy against COVID-19 disease in clinical trials of children and young people (CYP). Consequently, we investigated the uptake, safety, effectiveness and waning of the protective effect of the BNT162b2 against symptomatic COVID-19 in CYP aged 12–17 years in Scotland.

**Methods:**

The analysis of the vaccine uptake was based on information from the Turas Vaccination Management Tool, inclusive of Mar 1, 2022. Vaccine safety was evaluated using national data on hospital admissions and General Practice (GP) consultations, through a self-controlled case series (SCCS) design, investigating 17 health outcomes of interest. Vaccine effectiveness (VE) against symptomatic COVID-19 disease for Delta and Omicron variants was estimated using a test-negative design (TND) and S-gene status in a prospective cohort study using the Scotland-wide Early Pandemic Evaluation and Enhanced Surveillance of COVID-19 (EAVE II) surveillance platform. The waning of the VE following each dose of BNT162b2 was assessed using a matching process followed by conditional logistic regression.

**Findings:**

Between Aug 6, 2021 and Mar 1, 2022, 75.9% of the 112,609 CYP aged 16–17 years received the first and 49.0% the second COVID-19 vaccine dose. Among 237,681 CYP aged 12–15 years, the uptake was 64.5% and 37.2%, respectively. For 12–17-year-olds, BNT162b2 showed an excellent safety record, with no increase in hospital stays following vaccination for any of the 17 investigated health outcomes. In the 16–17-year-old group, VE against symptomatic COVID-19 during the Delta period was 64.2% (95% confidence interval [CI] 59.2–68.5) at 2–5 weeks after the first dose and 95.6% (77.0–99.1) at 2–5 weeks after the second dose. The respective VEs against symptomatic COVID-19 in the Omicron period were 22.8% (95% CI -6.4–44.0) and 65.5% (95% CI 56.0–73.0). In children aged 12–15 years, VE against symptomatic COVID-19 during the Delta period was 65.4% (95% CI 61.5–68.8) at 2–5 weeks after the first dose, with no observed cases at 2–5 weeks after the second dose. The corresponding VE against symptomatic COVID-19 during the Omicron period were 30.2% (95% CI 18.4–40.3) and 81.2% (95% CI 77.7–84.2). The waning of the protective effect against the symptomatic disease began after five weeks post-first and post-second dose.

**Interpretation:**

During the study period, uptake of BNT162b2 in Scotland has covered more than two-thirds of CYP aged 12–17 years with the first dose and about 40% with the second dose. We found no increased likelihood of admission to hospital with a range of health outcomes in the period after vaccination. Vaccination with both doses was associated with a substantial reduction in the risk of COVID-19 symptomatic disease during both the Delta and Omicron periods, but this protection began to wane after five weeks.

**Funding:**

UK Research and Innovation (Medical Research Council); Research and Innovation Industrial Strategy Challenge Fund; Chief Scientist's Office of the Scottish Government; Health Data Research UK; National Core Studies – Data and Connectivity.

**Research in context**

**Evidence before this study**

We searched PubMed on March 31, 2022, using the search terms “(COVID-19 OR SARS-CoV-2) AND vaccin* AND (children OR adolescent* OR young people)” with no restriction on language. Among 4,723 studies, we found 66 titles relevant to our areas of interest, namely vaccine uptake, safety, effectiveness, or waning. From those studies, it was clear that the two-dose BNT162b2 vaccine safety, immunogenicity and efficacy against COVID-19 in CYP has been initially demonstrated by the phase 3 clinical trials sponsored by the manufacturers – firstly those aged 16–17, then 12–15, and most recently 5–11 years. In China, Israel, the United States and England, the uptake of the first dose among 12–17 year-olds was typically above 50%, while it was slightly lower for the second dose and in younger age groups. The analyses of safety reported myocarditis as a rare outcome in high-school boys following the second dose of the BNT162b2 vaccine. Sleep irregularities were also reported as a possible adverse effect. The effectiveness of the first dose against symptomatic COVID-19 disease for the Delta variant in 12–17-year-olds typically ranged between 55–65% and of the second dose between 87–99%. The effectiveness against more severe outcomes requiring hospitalisation was better than against symptomatic infections. A few reports on VE against the Omicron variant suggested that VE is considerably lower in comparison to the Delta variant. Analysis of the waning of the protective effect showed a relatively rapid decline in effectiveness against symptomatic disease, within only a few weeks. VE against severe forms of COVID-19 may last longer, but this still needs to be confirmed.

**Added value of this study**

To our knowledge, this is the first study of COVID-19 vaccine uptake, safety, VE and waning against symptomatic disease after two doses for an entire nation, addressing both the Delta and the Omicron variant period. Among 16–17 year-olds in Scotland, 75.9% received the first dose and 49.0% the second dose; among 12–15 year-olds, the uptake was 64.5% and 37.2%, respectively. The safety record was encouraging, with none of the 17 investigated health outcomes showing an increased rate of hospitalisation following vaccination. In the 16–17-year-old group, VE against symptomatic COVID-19 during the Delta period was 64.2% (95% confidence interval [CI] 59.2–68.5) at 2–5 weeks after the first dose and 95.6% (77.0–99.1) at 2–5 weeks after the second dose. The respective VEs against symptomatic COVID-19 in the Omicron period were 22.8% (95% CI -6.4–44.0) and 65.5% (95% CI 56.0–73.0). In children aged 12–15 years, VE against symptomatic COVID-19 during the Delta period was 65.4% (95% CI 61.5–68.8) at 2–5 weeks after the first dose, with no observed cases at 2–5 weeks after the second dose. The corresponding VE against symptomatic COVID-19 during the Omicron period were 30.2% (95% CI 18.4–40.3) and 81.2% (95% CI 77.7–84.2). The waning of the protective effect started after five weeks post-first and post-second doses.

**Implications of all the available evidence**

High levels of BNT162b2 coverage can be achieved in CYP, with subsequent effectiveness against symptomatic COVID-19. However, protection against symptomatic disease is short-lived. We found no increase in hospitalisation following vaccination for any of the adverse health outcomes of interest.

Alt-text: Unlabelled box

## Introduction

The early research to characterise SARS-CoV-2 infection and COVID-19 disease in children and young people (CYP) was initially focused on the clinical presentation, patterns of spread, viral load, diagnosis and treatment, and the arguments related to vaccination of CYP which we recently reviewed^1^^,^^2^ – see also Box 1.^3^, ^4^, ^5^, ^6^, ^7^, ^8^, ^9^, ^10^, ^11^, ^12^, ^13^, ^14^, ^15^, ^16^, ^17^, ^18^, ^19^, ^20^, ^21^, ^22^, ^23^, ^24^, ^25^, ^26^, ^27^, ^28^, ^29^, ^30^, ^31^, ^32^, ^33^, ^34^, ^35^, ^36^, ^37^, ^38^, ^39^ CYP had milder symptoms, but research efforts focused on protecting particularly vulnerable children.^1^ After the first vaccines were licensed for the adult population, a complex discussion on an opportunity for vaccinating CYP arose, but low risks of severe outcomes and hospitalisation in CYP made the public debate on vaccination of CYP complex and at times polarised.^2^ In Scotland, COVID-19 vaccination was offered to all eligible 16–17 year-olds from Aug 6, 2021,^40^ then to all 12–15 year-olds from Sep 20, 2021,^41^ while 5–11 year-olds were offered a vaccine from Mar 19, 2022.^42^^,^^43^ (Supplementary Table S1). The two-dose BNT162b2 mRNA (henceforth BNT162b2) was the only licensed vaccine for CYP in the UK until March 2022. Initially, the general population of 12–17 year-olds were only offered a single dose of vaccine, but a second dose was recommended by the Joint Committee on Vaccination and Immunisation (JCVI) in Nov 2021^40^^,^^43^

**Box 1:** Background information on vaccination of CYP to prevent COVID-19 and the related research on vaccine effectiveness and safety conducted to date.

Following the first clinical trials, the arguments in favour of vaccination of CYP were excellent initial results on safety, immunogenicity and efficacy of the two-dose BNT162b2 vaccine against COVID-19 disease and-or symptomatic infection in CYP. They have been initially demonstrated by the phase 3 clinical trials sponsored by the manufacturers – firstly those aged 16–17, then 12–15, and then 5–11 years.^3^, ^4^, ^5^, ^6^, ^7^, ^8^, ^9^ Since then, tracking the uptake, monitoring safety, and analysing the vaccine effectiveness (VE) and waning in the “real world” context have emerged as policy priorities.^10^ The first reports were published by the research groups from China, Israel, the United States and the UK (for England only). In those countries, the uptake of the first dose among the 12–17 year-olds was typically above 50%, while it was slightly lower for the second dose and in younger age groups.^11^^,^^12^ The safety record was excellent to date, with rare myocarditis in boys aged 12–18 years following mainly the second dose of the BNT162b2 vaccine and sleep irregularities/disturbances being consistently reported as the main serious adverse effects among CYP.^13^, ^14^, ^15^, ^16^, ^17^, ^18^ A recent study of 0.4 million adolescents in South Korea suggested the absolute risk for myocarditis and/or pericarditis of 1.8 per 100,000 among first-dose recipients and 4.3 per 100,000 in second-dose recipients; furthermore, among 2.8 million adolescents aged 12–17 in the USA, a risk of serious adverse effects following the third (booster) dose was 2.7 per 100.000.^19^^,^^20^ Among 5–11 year-olds, the reported risk of serious post-vaccination adverse effects following two doses is even smaller, about 1.1 per 100,000.^21^ The effectiveness of the first dose against symptomatic COVID-19 disease for the Delta variant in the 12–17 years age group typically ranged between 55–65%, and of the second dose between 87–99%.^19^^,^^22^, ^23^, ^24^, ^25^, ^26^, ^27^ The VE against more severe outcomes that required hospitalisation was typically even higher.^28^^,^^29^ Reports on VE against Omicron are still rare and they suggest that VE is considerably smaller than against Delta.^30^, ^31^, ^32^ Analysis of the waning of the protective effect showed a relatively rapid decline in effectiveness against symptomatic disease, within several weeks of vaccination.^33^^,^^34^ VE against severe forms of the COVID-19 that require hospitalisation, as well as against death, lasts longer but also wanes gradually.^33^^,^^34^ In the UK, the policy decisions on vaccinating CYP were driven by scientific evidence on the health risks and benefits of the vaccine in CYP, which took time to obtain, and the consideration of the positive impact on school attendance and overall well-being of children.^35^, ^36^, ^37^ In England, the first dose VE in 12–15-year-olds caused by Delta variant peaked at 74.5% between 2–3 weeks after vaccination but then declined gradually to 45.9% after 10–12 weeks. For Omicron, the peak VE after the first dose in this age group was 49.6%, but then dropped to 16.1% in respective time periods post-vaccination. However, after the second dose, VE increased to 93.2% for Delta and 83.1% for Omicron. For 16–17-year-olds in England, respective VE after the first dose was 75.9% for Delta variant and then it declined to 29.3%, while for Omicron the peak post first dose reached 52.7% and then fell to 12.5%. The second dose increased VE to 96.1% for Delta and 76.1% for Omicron, but fell to 22.6% for Omicron while it continued to hold for Delta at 83.7%. This is consistent with the reports from the USA and Israel to date.^19^^,^^22^, ^23^, ^24^, ^25^, ^26^, ^27^ The Vaccine Adverse Event Reporting System (VAERS) is the national vaccine safety monitoring system in the United States that accepts reports of adverse events after vaccination.^38^ A study of VAERS reports relating to BNT162b2 vaccination in 12–17 year-olds in the USA from Dec 14, 2020 to July 16, 2021 found that the most commonly reported conditions and diagnostic findings among reports of serious events were consistent with a diagnosis of myocarditis (chest pain (56.4%), increased troponin levels (41.7%), myocarditis (40.3%), increased C-reactive protein (30.6%), and negative SARS-CoV-2 test results (29.4%)).^38^ V-safe is a smartphone-based tool that uses text messaging and web surveys to provide personalised health check-ins after COVID-19 vaccination which helps CDC monitor the safety of COVID-19 vaccines in near real-time.^39^ Studying v-safe data from Dec 14, 2020 to Jul 16, 2021 found that fewer than 1% of adolescents aged 12–17 years required medical care for any reason in the week after receipt of either dose, with only 0.04% hospitalised.^39^

We developed a protocol for evaluating the “real-world” effectiveness of the COVID-19 vaccination programme.^44^ Our aims in relation to vaccination of CYP in Scotland were to: (i) describe the uptake of the first and second doses in 16–17- and 12–15-year-olds; (ii) evaluate the safety of the vaccine after each dose; (iii) assess vaccine effectiveness (VE) against symptomatic disease caused by Delta and Omicron variants in both age groups; and (iv) investigate vaccine effectiveness waning.

## Methods

Information on the cohorts used for each analysis, primary data sources, data diagram and key dates relevant to the time period of different analyses are presented in Supplementary Table S2 and Supplementary Figures S1–S6.^45^, ^46^, ^47^, ^48^

### Vaccine uptake

The analysis of the vaccine uptake was based on information gathered from the Turas Vaccination Management Tool, inclusive of the date Mar 1, 2022 (Table 1). The number of CYP in the denominator (N=350,300) reflected the number of CYP in the Early Pandemic Evaluation and Enhanced Surveillance of COVID-19 (EAVE II) cohort when it was established.^45^ Vaccine uptake figures in this paper were based on a cross-sectional uptake among children aged 12–17 linking into EAVE II on March 2021, who were vaccinated by March 01, 2022. These will slightly differ from those published on the Public Health Scotland (PHS) website, which reports cumulative vaccine uptake by the age of the children at the date of vaccination.

**Table 1:** Uptake of BNT162b2 vaccine among 16–17 and 12–15 year-old children and young people in Scotland: status at a date Mar 1, 2022.

		16–17 years (from Aug 6th, 2021)			12–15 years (from Sep 20th, 2021)		
Dose	Sex	Number vaccinated	Population	% Uptake	Number vaccinated	Population	% Uptake
First dose	Females	42,545	54,919	77.5	75,813	116,420	65.1
	Males	42,891	57,690	74.3	77,568	121,261	64.0
	Total	85,436	112,609	75.9	153,381	237,681	64.5
Second dose	Females	27,770	54,919	50.6	43,812	116,420	37.6
	Males	27,364	57,690	47.4	44,793	121,261	36.9
	Total	55,134	112,609	49.0	88,605	237,681	37.2

Uptake figures include 6,865 CYP vaccinated before the period of the study (i.e., Aug 6, 2021). This is a sub-sample of CYP with specific individual or household vulnerabilities, who were offered vaccination before these dates and also followed a different schedule of recommended follow-up doses (detailed in Supplementary Table S2). Vulnerabilities included conditions such as a history of chronic respiratory disease, chronic heart conditions, chronic conditions of the kidney, liver or digestive system, chronic neurological disease, endocrine diseases, dysfunction of the spleen, serious genetic abnormalities, pregnancy, and different forms of immunosuppression (detailed in Supplementary Tables S2 and S3).

### Vaccine safety

The safety of the BNT162b2 vaccine in CYP was evaluated using national data on hospital admissions and General Practice (GP) consultations, through a self-controlled case series (SCCS) design. This analysis is not restricted to EAVE II data, and it includes all vaccinated 12–17-year-olds in Scotland, including those who were at higher risk of developing severe COVID-19. More than 95% of children aged 12–17 years are included in the EAVE II data, and the missing ones are likely to be new arrivals in Scotland. Twenty-nine potential adverse events of special interest (AESI) were chosen for inclusion in this study based on the list of outcomes recommended for monitoring by the World Health Organization,^49^ Safety Platform for Emergency vACcines (SPEAC),^50^ those previously monitored for influenza vaccinations,^51^ and through discussions with clinicians within PHS and the University of Edinburgh. The 29 AESI were grouped into 17 health outcomes by clinicians within PHS according to similarities in disease processes and outcomes (presented in Supplementary Table S4). We examined the association of exposures with hospital admissions for poisoning in CYP as a negative control outcome, which is assumed not to be associated with exposure to vaccination or SARS-CoV-2 infection. Each health outcome was defined using the International Classification of Diseases-10 diagnostic codes (ICD-10).^52^ Patients aged 12–17 years admitted to the hospital with an AESI diagnosis were identified using the Scottish Morbidity Record 01 (SMR01) national admission dataset.^53^ (details in Supplementary Tables S5 and S6).

The number of hospital stays that occurred in a baseline period (75-to-15-days before the first dose BNT162b2 vaccination, and during defined risk periods following vaccination) were calculated for each individual, for each vaccine dose number and health condition (Table 2). A SCCS analysis was undertaken to study the temporal association between the first and second dose BNT162b2 and 17 health outcomes in 12–17-year-olds in Scotland. Incidence rate ratios (IRRs) were estimated to quantify the rate of hospital stays for a health outcome in the risk period following vaccination relative to the baseline period. All hospital stays in the periods are included to study both incident cases and exacerbations of existing conditions. An IRR>1 suggests an increased rate of hospitalisation following vaccination. The SCCS analysis was only conducted for outcomes where at least 5 hospital stays were recorded in the risk period following vaccination for a given health outcome. These methods were used in an additional analysis of GP consultations for myocarditis and pericarditis as the health outcome, as these were the most frequently reported outcomes of concern in previous studies on vaccine safety.^13^, ^14^, ^15^, ^16^, ^17^, ^18^ All GP consultations with a recorded read code associated with myocarditis and/or pericarditis were included in the analysis. Detailed methods for the SCCS analysis are presented in Supplementary Table S5.

**Table 2:** The number of hospital stays for each health outcome in the risk periods following the first and the second dose BNT162b2 vaccine. (Hospital stay counts N<5 have been suppressed in accordance with statistical disclosure procedures; day of vaccination is day 0; baseline period runs from the day (−75) to the day (−15); numbers in brackets=number of individual subjects contributing to the total number of hospital stays).

Health outcome and risk period (days)	Number of hospital stays following the first dose	Number of hospital stays following the second dose
Type 1 diabetes		
1 – 90	92 (80)	46 (37)
Vasculitis and inflammatory conditions		
1 – 42	22 (<5)	11 (<5)
Seizures		
0 – 6	13 (11)	19 (16)
Chronic fatigue		
1 – 90	9 (9)	0
Demyelination		
1 – 42	<5	<5
1 – 90	<5	<5
Thrombocytopenia		
1 – 21	<5	<5
1 – 42	<5	<5
Arthritis		
1 – 42	<5	<5
1 – 90	<5	<5
Neuropathy, encephalitis and myelitis		
1 – 7	<5	0
1 – 42	<5	0
1 – 90	<5	<5
Narcolepsy		
1 – 42	<5	0
Thrombosis and embolism		
1 – 21	0	<5
1 – 42	<5	<5
Haemorrhagic stroke		
1 – 21	0	<5
1 – 42	<5	<5
Autoimmune thyroiditis		
1 – 42	0	0
1 – 90	<5	0
Myocarditis and pericarditis		
1 – 42	0	<5
Anaphylaxis		
0 – 1	0	0
Guillain-Barre syndrome		
1 – 42	0	0
1 – 90	0	0
Myasthenia gravis		
1 – 42	0	0
Disseminated intravascular coagulation		
1 – 21	0	0
1 – 42	0	0

### Vaccine effectiveness and waning

To study VE and examine waning, we used the Early Pandemic Evaluation and Enhanced Surveillance of COVID-19 database comprising linked vaccination, primary care, and reverse transcriptase-polymerase chain reaction (RT-PCR) testing for children and young people (aged 16–17 and 12–15 years) in Scotland.^45^, ^46^, ^47^, ^48^ The endpoint of the analysis was symptomatic COVID-19 disease with RT-PCR test positivity for SARS-CoV-2 infection.

The estimates are based upon a national prospective cohort study with test negative cohort design, where vaccination records are linked to Electronic Communication of Surveillance in Scotland (ECOSS) for an endpoint of COVID-19 RT-PCR positive test and a notion of the presence of symptoms. Symptom status is based upon self-reported information provided at the time of completion of the online test request form. Details on ECOSS data are available in Supplementary Table S2.

In this study, two periods were defined based on the dominant SARS-CoV-2 variant circulating in communities at the time, Delta and Omicron.^48^ The “Delta period” was defined as Aug 6, 2021, to Dec 19, 2021; the “Omicron period” ranged from Dec 20, 2021, to Apr 18, 2022.^48^ SARS-CoV-2 test results were assigned to each period as follows: test-negative design (TND) analyses used a combination of SARS-CoV-2 RT-PCR test results and S-gene target PCR results to determine the Delta and Omicron periods. The Delta variant and the BA.2 Omicron variant are S-gene positive whereas the Omicron BA.1 is S-gene negative. S-gene presence and absence can be used as a proxy for variant type. SARS-CoV-2 RT-PCR positive tests between Aug 6, 2021, and Nov 1, 2021, were considered the Delta variant. Between Nov 1, 2021, and Jan 15, 2022, S-gene PCR results were used to determine variants as either Delta or Omicron BA.1. Any PCR positive tests without an S-gene PCR result during this time period were discarded and their fraction was indeed minimal (see Supplementary Figure S6). SARS-CoV-2 RT-PCR positive tests between Jan 15, 2022, and Apr 18, 2022, were considered Omicron (BA.1 or BA.2). Supplementary Figures S4–S6 show the proportion of RT-PCR tests in all tests conducted among 12–17-year-olds during the study period and the proportion of RT-PCR tests that had to be discarded after Nov 1, 2021, because of the absence of S-gene information. Supplementary Figure S4 also includes information on the number of lateral flow tests, which were used in schools, but which were not analysed in this study (see Discussion section).

This analysis includes the BNT162b2 vaccine for two doses. In Scotland, all children were on a 2-dose schedule, apart from immunocompromised, who are on a 3-dose schedule. A matched test-negative design (TND) was used to estimate the odds ratios comparing unvaccinated at the time of test with post-first and post-second dose vaccination periods measured in weeks. Positive tests were matched with up to 5 controls using the date of test, age and location, with a conditional logistic regression model stratified by the positive case and individually matched controls. Positive tests which could not be matched on these variables were discarded. The conditional logistic regression model included vaccine status, sex, socioeconomic status measured by quintiles of the Scottish Index of Multiple Deprivation (SIMD), whether the CYP had previously tested positive at any time before the specimen date, the number of tests previously taken, and the following QCovid risk conditions: Blood Cancer, Cerebral Palsy, Epilepsy, Fracture, Severe Mental Illness, Learning Disability, Congenital Heart Defect, and the number of other QCovid risk conditions the individual has. VEs were measured relative to the unvaccinated. Vaccine effectiveness is defined as “1 – odds ratio” from the conditional logisitic regression. Details on statistical modelling are presented in Supplementary Table S2. They include the full set of variables used to analyse the waning of vaccine effectiveness.

### Reporting and availability of code

We provided STROBE and RECORD statements in Supplementary Table S7. The availability of data and code is explained in the acknowledgements.

### Patient and public involvement

The EAVE II Public Advisory Group reviewed the analysis design for this project. Among their suggestions relevant to this work, we complied by explaining any excluded groups or aggregations should and separately reporting vaccine uptake in 16–17 year-olds.

### Role of the funding source

The funders did not have any influence on the study design, analyses, interpretation or decision to publish.

## Results

### Vaccine uptake

**Figure 1:**
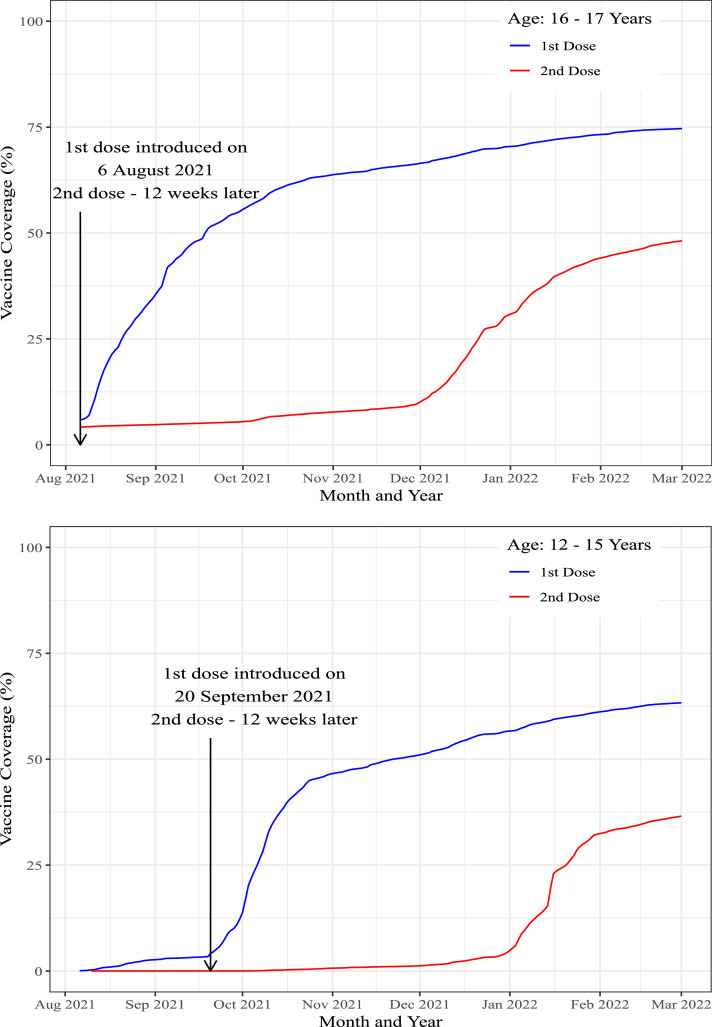
Visualisation of the uptake of BNT162b2 vaccine among 16-17 (left graph) and 12-15 (right graph) year-olds in Scotland: blue lines represent first dose uptake, red lines second dose uptake. Status at a date Mar 1, 2022.

There were 350,300 CYP aged between 12–17 years who were resident in Scotland during the period of study between Aug 6, 2021 and Mar 1, 2022 (Table 1). The 16–17-year-old group was the first to receive the vaccine. As of Mar 1, 2022, 74.3% of males and 77.5% of females have received their first dose, and 47.4% of males and 50.6% of females the second dose (Figure 1). Among 12-15-year-olds, the uptake of the first dose reached 64.0% in males and 65.1% in females, and of the second dose 36.9% in males and 37.6% in females.

### Vaccine safety

Among 12–17-year-olds, 13 of the 17 studied health outcomes had fewer than five hospital stays in their corresponding risk periods following both first and second dose BNT162b2 vaccination (Table 2). For those conditions five or more stays, a SCCS analysis was undertaken. 92 hospital stays were recorded for type 1 diabetes in the 1–90 days following the first dose and 46 following the second dose (Table 2), but no significant increase in the rate of hospital stays following vaccination was found (post-first dose IRR: 0.74, 95% CI 0.54-1.02; post-second dose IRR: 0.73, 95% CI 0.49-1.09) (Supplementary Table S5). A total of 22 hospital stays were recorded for vasculitis and inflammatory conditions in the 1–42 days following the first dose and 11 following the second dose (Table 2), but no significant increase in the rate of hospital stays following vaccination was found (post-first dose IRR: 2.15, 95% CI 0.90-5.12; post-second dose IRR: 2.75, 95% CI 0.81-9.30). Note that the number of individuals contributing to the total number of hospital stays for vasculitis and inflammatory conditions following the first and second dose was <5 in both cases. A total of 13 hospital stays for seizures were recorded in the 0–6 days following the first dose and 19 following the second dose (Table 2), but no significant increase in the rate of hospital stays following vaccination was found (post-first dose IRR: 0.71, 95% CI 0.40-1.27; post-second dose IRR: 1.25; 95% CI 0.75-2.07) (Supplementary Table S5). There were also 9 recorded hospital stays for chronic fatigue in the 1–90 days following the first dose and none after the second dose (Table 2), but no significant increase in the rate of hospital stays following first dose vaccination was found (IRR: 1.71, 95% CI 0.49-5.91) (Supplementary Table S5). No hospital stays for myocarditis or pericarditis were recorded in the 1–42 days following the first dose and <5 following the second dose (Table 2). Given that myocarditis and pericarditis were the most widely reported concerns in previous studies,^13^, ^14^, ^15^, ^16^, ^17^, ^18^ we conducted an additional SCCS study based on GP consultations related to myocarditis and pericarditis instead of hospital stays. There were five GP consultations for myocarditis and pericarditis following the first dose in this period, and <5 following the second dose. No significant increase in the rate of GP consultations following first dose vaccination was found (IRR: 1.61, 95%CI 0.29-8.84; *p*=0.070) (Supplementary Table S5). We examined the associations of exposures with hospital admissions for poisoning in CYP as a negative control outcome. We found no increased risk of poisoning in the 1–90 days following vaccine exposure (Supplementary Tables S4 and S5).

### Vaccine effectiveness and waning of the protective effect

**Figure 2:**
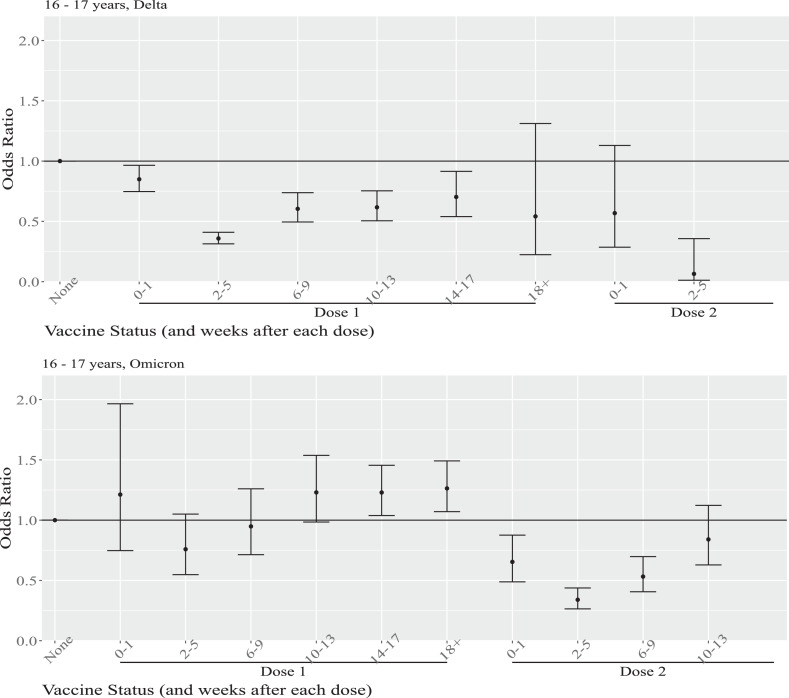

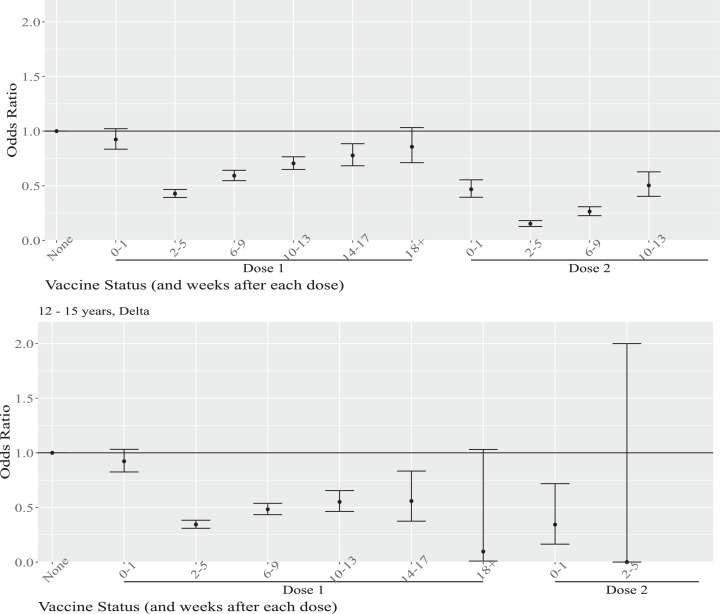
**a-d:** (a) top left: vaccine effectiveness (VE) of the BNT162b2 vaccine in 16-17 year olds, expressed as risk ratios (Y-axis) and weeks after each dose (X-axis), for the Delta variant; (b) top right: vaccine effectiveness for the BNT162b2 vaccine in 16-17 year olds, for the Omicron variant; (c) bottom left: vaccine effectiveness for the BNT162b2 vaccine in 12-15 year olds, for the Delta variant; (d) bottom right: vaccine effectiveness (VE) of the BNT162b2 vaccine in 12-15 year olds, for the Omicron variant. Odds ratios were estimated using a conditional logistic regression model, with positive tests matched to negative controls by age, location, and date of test. The model contains adjustments for sex, deprivation, urban/rural classification, previous positivity, number of previous tests and risk group category.

For the Delta period, among 16–17-year-olds the first dose led to a 64.2% (95% CI 59.7-68.5) reduction in symptomatic COVID-19 in the period 2–5 weeks after vaccination, but the effect waned to 34.8% (95% CI 20.9-46.2) after 10-13 weeks (Table 3, Figure 2a). However, VE increased to 95.6% (95% CI 77.0-99.1) in the period 2-5 weeks after the second dose, remaining at 96.7% (95% CI 51.3-99.8) in the period 6+ weeks after the second dose (Table 3, Figure 2a).

**Table 3:** Effectiveness of the BNT162b2 in 16–17 year-olds. Odds ratios are shown for Delta and Omicron periods. Odds ratios were estimated using a conditional logistic regression model, with positive tests matched to negative controls by age, location, and date of test. The model contains adjustments for sex, deprivation, urban/rural classification, previous positivity, number of previous tests and risk group category. (VE=vaccine effectiveness; L95%CI=lower 95% confidence limit; U95%CI=upper 95% confidence limit; “N” is the number of persons contributing to each sub-sample; “events” is the number of episodes of symptomatic infections with positive tests that were recorded among those persons in each sub-sample. Due to lack of events in the period post-second dose for the Delta period, we collapsed them all into a 6+ weeks category, while for the Omicron period we report categories 6–9, 10–13 and 14+ weeks categories).

		Delta Period				Omicron Period			
Vaccine Status (dose,week)	N	Events	VE%	L95%CI	U95%CI	Events	VE%	L95%CI	U95%CI
None	16722	3368				1365			
1^st^ 0–1	2927	615	13.0	1.4	23.3	60	−18.4	−89.3	26.0
1^st^ 2–5	4510	499	64.2	59.2	68.5	116	22.8	−6.4	44.0
1^st^ 6–9	1792	309	39.8	27.0	50.3	179	11.9	−16.1	33.1
1^st^ 10–13	2303	368	34.8	20.9	46.2	340	−22.4	−52.3	1.6
1^st^ 14–17	3319	253	28.7	8.0	44.8	790	−24.2	−46.5	−5.3
1^st^ 18+	2268	16	51.8	−16.4	80.1	866	−24.7	−46.7	−6.0
2^nd^ 0–1	812	21	38.8	−15.8	67.7	154	34.0	13.2	49.9
2^nd^ 2–5	1259	2	95.6	77.0	99.1	173	65.5	56.0	73.0
2^nd^ 6+/6–9	852	1	96.7	51.3	99.8	229	43.4	26.9	56.2
2^nd^ 10–13	645	0				234	8.9	−19.1	30.3
2^nd^ 14+	219	0				89	1.2	−49.3	34.6

In the 16–17 year olds, VE during the Omicron period was smaller than during the Delta period (Table 3, Figure 2b). The first dose had a VE of 22.8% (95% CI -6.4-44.0%) 2-5 weeks after vaccination. The second dose showed a VE of 65.5% (95% CI 56.0-73.0%) after 2-5 weeks, but the effect was reduced to 8.9% (95% CI -19.1-30.3) after 10-13 weeks.

Among 12-15 year-olds for the Delta period, the first dose led to a 65.4% (95% CI 61.5-68.8) reduction in the period 2-5 weeks after vaccination, but the effect waned and dropped to 44.5% (95% CI 34.1-53.3) after 10-13 weeks (Table 4, Figure 2c). However, no symptomatic cases were observed in the period 2-5 weeks after the second dose, suggesting a VE of 100%, which decreased to 92.9% (95% CI 42.5-99.1) in the period 10-13 weeks after the second dose (Table 4, Figure 2c).

**Table 4:** Effectiveness of the BNT162b2 in 12–15-year-olds. Odds ratios are shown for Delta and Omicron variants. Odds ratios were estimated using a conditional logistic regression model, with positive tests matched to negative controls by age, location, and date of test. The model contains adjustments for sex, deprivation, urban/rural classification, previous positivity, number of previous tests and risk group category. (VE=vaccine effectiveness; L95%CI=lower 95% confidence limit; U95%CI=upper 95% confidence limit; “N” is the number of persons contributing to each sub-sample; “events” is the number of episodes of symptomatic infections with positive tests that were recorded among those persons in each sub-sample. Due to lack of events in the period post second dose for the Delta period, we collapsed them all into a 6+ weeks category, while for the Omicron period we report categories 6–9, 10–13 and 14+ weeks categories).

		Delta period				Omicron period			
Vaccine Status (dose, week)	N	Events	VE%	L95%CI	U95%CI	Events	VE%	L95%CI	U95%CI
None	102169	18915				4776			
1^st^ 0–1	4164	882	8.0	−2.8	17.7	209	14.2	−10.3	33.2
1^st^ 2–5	7785	811	65.4	61.5	68.8	526	30.2	18.4	40.3
1^st^ 6–9	9589	1137	50.9	45.3	55.8	974	21.8	11.5	30.8
1^st^ 10–13	10892	455	44.5	34.1	53.3	2426	16.9	8.7	24.4
1^st^ 14–17	2975	55	47.5	22.1	64.6	895	9.5	−3.6	20.9
1^st^ 18+	1414	4	93.5	43.4	99.3	493	5.4	−13.4	21.0
2^nd^ 0–1	1926	14	62.4	20.2	82.3	392	46.9	37.0	55.3
2^nd^ 2–5	2710	0	100.0	−Inf	100.0	310	81.2	77.7	84.2
2^nd^ 6+/6–9	3155	1	92.9	42.5	99.1	613	68.5	63.4	72.9
2^nd^ 10–13	1050	0	0.0	0.0	0.0	270	43.3	30.0	54.2
2^nd^ 14+	226	0	0.0	0.0	0.0	65	48.7	22.0	66.3

When examining VE during the Omicron period in 12-15-year-olds, the first dose led to a 30.2% (95% CI 18.4-40.3) reduction 2-5 weeks after vaccination, but this effect waned to 16.9% (95% CI 8.7-24.4) after 10-13 weeks (Table 4, Figure 2d). The second dose increased VE to 81.2% (95% CI 77.7-84.2) after 2-5 weeks, but after 10-13 weeks the effect was reduced to 43.3% (95% CI 30.0-54.2).

## Discussion

This study showed that high levels of BNT162b2 vaccine coverage can be achieved among CYP through national vaccination programmes. Once licensed for 16–17-year-olds, the first dose of BNT162b2 reached coverage of above 50% within a month. However, the uptake was much slower thereafter, reaching 75.9% by Mar 1, 2022. A similar pattern was observed among the 12–15-year-olds, where the uptake was also rapid within the first month of the programme, but it eventually reached 64.5% and stalled. By the end date of this study, the uptake was lower for the second dose and among the younger age group at that point in time.

There are many possible contributing factors to this finding: some children may have not yet been eligible for their second dose before the end date of this study, due to the required interval between doses. This is particularly likely to apply to the younger age group who received their offer of the first dose at a later date, or the second dose may have been delayed due to symptomatic COVID-19 following the first dose. Other possible reasons include the initial offer being a single dose schedule potentially influencing the perception of the need for a second dose; then, the perceived rarity of severe illness in children; furthermore, there could have been increased awareness of reports of sub-optimal vaccine prevention of the spread of infection, of possible side-effects, and of the waning of the protective effect.^46^^,^^47^^,^^54^, ^55^, ^56^, ^57^

Vaccination is overall safe and effective in protecting against symptomatic COVID-19 disease the first few months post-vaccination. However, the protection against symptoms was short-lived during the Delta and Omicron periods in Scotland. In both 16–17 and 12-15 year-olds, the waning of the effectiveness during the Delta period was detectable from week 6 following the first dose. However, the effectiveness remained higher for a longer time period following the second dose. In both age groups, the effectiveness of the first dose during the Omicron period was modest and it waned rapidly after week 5. Furthermore, the second dose offered stronger and more durable protection, but it also typically waned from week 6 following the second dose. For adults, protection against severe outcomes was higher and longer-lasting than protection against symptomatic infection.^46^ It is likely that the same is also true for CYP infected with Omicron, but further research is required to confirm this.

The UK medicines regulator, the Medicines and Healthcare products Regulatory Agency (MHRA), has assessed the BNT162b2 vaccine and declared it safe for 12–17 year-olds. This followed a rigorous review of the safety, quality and effectiveness of these vaccines among CYP of that age.^58^ However, the safety of these vaccines is still subject to monitoring as the vaccine is rolled out to the population. The overall picture is reassuring, but there are some reports of potential side effects, including mild myocarditis and myopericarditis in young people (see Box 1).^14^^,^^38^^,^^39^^,^^47^^,^^59^ In terms of vaccine safety, we found no reasons for concern after studying hospitalisations. However, five GP consultations for myocarditis and/or pericarditis post-first dose in this period warrant further monitoring, particularly given reports from other countries about this side effect.^2^ All GP consultations with a recorded read code associated with myocarditis and/or pericarditis were included in the analysis. We cannot be certain whether these consultations would reflect possible, probable, or confirmed myocarditis or pericarditis diagnoses. The interpretation of those rare consultations should be carefully weighed against ascertainment bias, i.e. an increased likelihood to seek advice for these symptoms, and for clinicians to attribute compatible symptoms to these conditions, in the context of vaccination and reported complications.

In Scotland, there were very few hospitalisations for the 17 health outcomes in 12–17 year-olds following the first and second dose of the BNT162b2 (Table 2), with no recorded stays for myocarditis and pericarditis following the first dose, and < 5 following the second dose. This is consistent with a randomised, placebo-controlled, observer-blinded, phase 3 trial assessing the safety of BNT162b2 vaccination in healthy persons aged 12-15 years.^5^ No association was found between the first or second dose and hospital stays for type 1 diabetes, vasculitis and inflammatory conditions, seizures and chronic fatigue. However, due to the small number of events, further investigations on larger populations are merited, particularly for rare serious adverse events such as myocarditis and pericarditis. Hospital stays, in general, are uncommon in this age group, and so the ability to conduct SCCS analysis was limited.^60^^,^^61^ Further, no association was found between the first dose and GP consultations for myocarditis and pericarditis. The vasculitis and inflammatory conditions health outcome includes the condition Paediatric Inflammatory Multisystem Syndrome (PIMS). PIMS is a new condition that happens weeks after someone has had the virus that causes COVID-19.^62^ No significant increase in the rate of hospital stays for vasculitis and inflammatory conditions following vaccination were found.

Our study has several strengths. It is a national-level study. This ensured sufficiently large numbers to observe effects and patterns. Previous studies using the EAVE II platform have demonstrated the usefulness of integrated data from multiple sources and enabled precise analyses of the pandemic almost in real-time,^45^, ^46^, ^47^ which then informed policies.

There are some caveats and limitations specific to COVID-19 research in CYP. First, there was a sub-group of CYP in Scotland (n=6,865) who were in the category of particularly vulnerable and at-risk from COVID-19. They were vaccinated in parallel with adults in Scotland, and before most CYP, using vaccines that were licensed for adults at the time. Therefore, they were not comparable to other CYP because of different inherent risks of COVID-19 complications, different time periods, exposure to different SARS-CoV-2 variants, and likely smaller average number of possible exposures because they were shielded. They also received different vaccines and dosages. This is why we did not include this relatively small sub-group of CYP in the VE analyses, but we retained them for safety analyses.

Moreover, a recent previous SARS-CoV-2 infection may have delayed vaccination and impacted vaccine uptake.^57^ Vaccine uptake figures in this paper were based on a cross-sectional uptake among children aged 12–17 linking into EAVE II at a specific date and then getting a vaccine by March 1, 2022. For this reason, they slightly differ from the figures published on the Public Health Scotland website, which reports cumulative vaccine uptake by the age of the children at the date of vaccination. Therefore, those two sets of figures are not directly comparable, although the difference is unlikely to be substantial.

In the study of safety, a preceding hospitalization during the period 75-to-15-days before the first dose of vaccine may have influenced the timing to receive vaccination, and thereby bias the SCCS data, although this is mainly a theoretical concern as such events were rare. The use of negative controls in SCCS, both at exposure or outcome side, has been proven to be a valuable approach to evaluate potential biases and many assumptions required to run a reliable SCCS.^63^, ^64^, ^65^, ^66^ Injuries among CYP are a useful negative control outcome, with poisoning being the most frequent cause for admission in this age group, as identified by ICD-10 codes T36-T50 inclusive. These are distinct from the codes that should be used for complications of a medicine, so should be independent from any vaccine complications.

Another limitation is that the assumptions made to determine dominant variant time periods may incorrectly assign COVID-19 cases to a given variant, thus impacting vaccine effectiveness calculations. Also, it was not possible to distinguish between specific sub-variants within the Omicron variant because only a subset of samples was sent for Whole Genome Sequencing (WGS). Furthermore, the second dose of the vaccine in both 16–17-year-olds and 12-15-year-olds was given with a delay of several weeks after the first dose (see Figure 1). In parallel, Omicron started to rapidly replace Delta as the dominant variant in Scotland. This is why the numbers available for studying VE of the 2nd dose of the vaccine in preventing infection by the Delta variant fell rapidly, and these estimates have wide confidence intervals. The “depletion of susceptible” bias may have played a role in the final weeks of the analysis, with a reasonably high vaccine coverage and high prevalence of the Omicron variant.

The outcome of TND was defined by the positive RT-PCR test and a provision in the dataset that the disease was symptomatic in a given test-positive case. A potential caveat in choosing the study design was that children were also tested twice a week at their schools using lateral flow tests (LFT). We decided to exclude all LFT results from the analyses because children are more likely to report positive than negative tests which could have introduced bias.^67^ Still, it should be noted that an appreciable proportion of children have likely performed an RT-PCR test after they had a positive LFT, which is another testing-related caveat. A further caveat is lack of data on antibody levels against SARS-CoV-2 which could then be correlated with VE and waning. We cannot expect to have this information available in a national-based study of this size, but further research on smaller cohorts should explore the relationships between information on the antibody levels, VE, waning and clinical symptoms.

The negative VE estimates in the Omicron period could have several plausible explanations. First, this could be due to behavioural confounding, where vaccinated CYP begin to act more freely and take less caution. This is how they can eventually get more symptomatic infections, as a net result of modest vaccine protection against symptomatic infection, waning of the effectiveness, and their behaviour. Initially, the vaccine effect is still dominant, but as it wanes over time, the behaviour effect might cause negative VE. Second, at this stage of the pandemic it is plausible that there were more CYP who acquired natural immunity among the unvaccinated group than among the vaccinated. Natural immunity may be either more robust, more durable, or both, in comparison to vaccine induced immunity against the omicron variant, leading to negative VE over time.^68^

We also planned to study VE against severe COVID-19 outcomes.^69^ However, we were concerned that CYP hospitalisations during the period of study might have been predominantly “with” COVID-19, rather than “because of” COVID-19, leading to a potential for misclassification bias. Although the Public Health Scotland's weekly report estimated that 60% of hospital admissions in Scotland in December 2021 were “because of” rather than “with” COVID-19,^69^ that figure is relevant to all ages, so it is still possible that the ratio in CYP is quite different. To study this further, we presented Supplementary Table S6 which presents hospitalisations of 12–17-year-olds within 14 days from testing positive anywhere in Scotland. The total number of hospitalizations during the study period was 199 and many of the admitted CYP had comorbidities (35.7%). The most frequent hospitalisation time was “0-1 days” (76.8%). Only 12.6% CYP stayed in hospital for more than 2 days (Supplementary Table S6). That suggests that, in CYP, many hospitalisations may be “with” COVID-19, rather than “because of” COVID-19, preventing a meaningful analysis. The number of hospitalisations truly due to COVID-19 and other severe outcomes was too small in Scotland over the period of study to support a conventional study design and lead to an interpretable result.^70^ Data from countries with larger child populations will provide better estimates on protection against severe outcomes for 12–17-year-olds, such as recent examples from England and Brazil.^30^^,^^71^

In this paper, we addressed several important research questions on CYP in a single publication. We needed to use different datasets and different study designs to address each of the posed research questions. The EAVE II collaboration has already published a number of separate research papers on uptake,^72^ safety,^73^ VE^46^^,^^74^, ^75^, ^76^, ^77^ and waning^54^ among the adults in Scotland. In those papers, specific aspects of studying each of those research questions, related to study design, biases, confounding and chance effects, were already discussed in greater depth, and they also apply in this study.

This study has several implications for policy, practice and research. So far, the achieved rates of uptake are high, but the rollout of vaccines to CYP started considerably later than in the adults. The study also showed that the BNT162b2 vaccine in children is both safe and effective in preventing symptomatic infection, but also that this protection rapidly wanes. This complicates the decisions for both the authorities, parents and their children. The most recent studies have confirmed our observations of limited effectiveness and relatively rapid waning of the 2-dose BNT162b2 COVID-19 vaccine in CYP 12–17 years of age.^78^^,^^79^ However, they demonstrated a considerable VE of the first booster dose, meaning that boosters in CYP may still be considered as an option to help maximise school attendance and reduce education disruption.^78^^,^^79^ Lack of information on the booster doses is, therefore, a limitation of our study.

There is a clear incentive to enable children to safely return to school and that children's regular school attendance is ensured.^80^^,^^81^ Vaccines could help this goal to an extent, and several studies demonstrated the effective use of health education campaigns to design and implement policies to overcome vaccine hesitancy among the parents and adolescents.^2^^,^^82^, ^83^, ^84^, ^85^ However, this study neither investigated if they had an impact on school attendance, nor whether the level of ‘symptomatic’ illness would have led to absence through symptom severity alone. In the current era of testing and isolation guidance, it is possible that many of the cases wouldn't be detected and that children would continue to attend school. Furthermore, any potential effect of vaccines on increased school attendance would, given the evidence of waning found here, only provide protection against symptomatic COVID-19 to children for the duration of up to one school term, posing challenges to policymakers. Education disruption is only one element of the rationale for vaccinating CYP. Critical to any benefit in this regard is any effect on transmission, which still needs to be better understood. Further research will be needed to clarify whether the protection against severe forms of the disease may, in fact, be higher and last longer than against the symptomatic infection.

## Contributors

A.S., C.R. and I.R. conceived the idea for this study. Analysis was undertaken by T.M. and K.A. IR wrote the draft of the paper and compiled supplementary online materials. Z.G., L.F., C.S., A.B., R.W., L.W., O.V.S., J.L.K.M., L.A.C., E.M., F.H., F.A., J.McM., U.A., S.A.S., S.K., C.R.S., S.V.K., and L.D.R. critically revised the manuscript, added comments and approved the final version for submission. C.R., T.M. and K.A. accessed and verified all the data. AS was responsible for the decision to submit the manuscript.

## Data sharing statement

A data dictionary covering the datasets used in this study can be found at https://github.com/EAVE-II/EAVE-II-data-dictionary. All code used in this study is publicly available at https://github.com/EAVE-II/Covid-Vaccines-CYP. The data used to undertake this analysis are not publicly available because they are based on deidentified national clinical records. These data are available, subject to approval by the NHS Scotland Public Benefit and Privacy Panel, by application through the Scotland National Safe Haven. The data used in this study are sensitive and will not be made publicly available.

## Declaration of interests

A.S., C.R. and J.McM. are members of the Scottish Government Chief Medical Officer's COVID-19 Advisory Group and A.S. of its Standing Committee on Pandemics. A.S. & J.McM. are also members of the New and Emerging Respiratory Virus Threats Advisory Group (NERVTAG) Risk Stratification Subgroup. J.McM. is the Chair of the multidisciplinary Scottish COVID-19 National Incident Management Team. A.S. is a member of AstraZeneca's Thrombotic Thrombocytopenic Taskforce. SVK is a member of the U.K. Government's Scientific Advisory Group on Emergencies subgroup on ethnicity, the Cabinet Office's International Best Practice Advisory Group and was co-chair of the Scottish Government's Expert Reference Group on Ethnicity and COVID-19. C.R. reports grants from the MRC and Public Health Scotland, during the conduct of the study, and is a member of the Scientific Pandemic Influenza Group on Modelling, Medicines and Healthcare products Regulatory Agency, Vaccine Benefit and Risk Working Group. I.R. is a member of the Scientific Council on COVID-19 of the Republic of Croatia and co-Editor-in-Chief of the Journal of Global Health. All roles have been unremunerated. All other authors report no potential competing interest.

## Appendix Supplementary materials

Image, application 1
